# Safety and efficacy of stereotactic body radiotherapy as primary treatment for vertebral metastases: a multi-institutional analysis

**DOI:** 10.1186/s13014-014-0226-2

**Published:** 2014-10-16

**Authors:** Matthias Guckenberger, Frederick Mantel, Peter C Gerszten, John C Flickinger, Arjun Sahgal, Daniel Létourneau, Inga S Grills, Maha Jawad, Daniel K Fahim, John H Shin, Brian Winey, Jason Sheehan, Ron Kersh

**Affiliations:** Department of Radiation Oncology, University of Wuerzburg, Wuerzburg, Germany; Department Radiation Oncology, University of Pittsburgh Medical Center, Pittsburgh, PA USA; Department of Neurological Surgery, University of Pittsburgh Medical Center, Pittsburgh, PA USA; Department of Radiation Oncology, Sunnybrook Odette Cancer Centre, Toronto, Canada; Department of Radiation Oncology, Princess Margaret Hospital, Toronto, Canada; Department of Radiation Oncology, Beaumont Health System, Oakland University William Beaumont School of Medicine, Royal Oak, MI USA; Department of Neurosurgery, Beaumont Health System, Oakland University William Beaumont School of Medicine, Royal Oak, MI USA; Department of Neurosurgery, Massachusetts General Hospital, Boston, MA USA; Department of Radiation Oncology, Massachusetts General Hospital, Boston, MA USA; Department of Neurological Surgery, University of Virginia School of Medicine, Charlottesville, VA USA; Riverside Medical Center, Newport News, VA USA; Department of Radiation Oncology, University of Zurich, Zurich, Switzerland

## Abstract

**Purpose:**

To evaluate patient selection criteria, methodology, safety and clinical outcomes of stereotactic body radiotherapy (SBRT) for treatment of vertebral metastases.

**Materials and methods:**

Eight centers from the United States (n = 5), Canada (n = 2) and Germany (n = 1) participated in the retrospective study and analyzed 301 patients with 387 vertebral metastases. No patient had been exposed to prior radiation at the treatment site. All patients were treated with linac-based SBRT using cone-beam CT image-guidance and online correction of set-up errors in six degrees of freedom.

**Results:**

387 spinal metastases were treated and the median follow-up was 11.8 months. The median number of consecutive vertebrae treated in a single volume was one (range, 1-6), and the median total dose was 24 Gy (range 8-60 Gy) in 3 fractions (range 1-20). The median EQD2_10_ was 38 Gy (range 12-81 Gy). Median overall survival (OS) was 19.5 months and local tumor control (LC) at two years was 83.9%. On multivariate analysis for OS, male sex (p < 0.001; HR = 0.44), performance status <90 (p < 0.001; HR = 0.46), presence of visceral metastases (p = 0.007; HR = 0.50), uncontrolled systemic disease (p = 0.007; HR = 0.45), >1 vertebra treated with SBRT (p = 0.04; HR = 0.62) were correlated with worse outcomes. For LC, an interval between primary diagnosis of cancer and SBRT of ≤30 months (p = 0.01; HR = 0.27) and histology of primary disease (NSCLC, renal cell cancer, melanoma, other) (p = 0.01; HR = 0.21) were correlated with worse LC. Vertebral compression fractures progressed and developed de novo in 4.1% and 3.6%, respectively. Other adverse events were rare and no radiation induced myelopathy reported.

**Conclusions:**

This multi-institutional cohort study reports high rates of efficacy with spine SBRT. At this time the optimal fractionation within high dose practice is unknown.

## Introduction

A single fraction of conventional radiotherapy with 8 Gy has been recommended for painful vertebral metastases [1–3]. However, this conventional radiotherapy is associated with only short term pain relief of 3 – 6 months. This might be sufficient for metastatic patients with short life expectancy. However, today validated scores are available to select a subgroup of patients with longer overall survival [4]. In parallel, improvements of overall survival due to more effective systemic treatments in many cancer types motivated the evaluation of radiation technology to maximize pain control and local control for the long term. With image guidance (IGRT), intensity modulated radiotherapy (IMRT), precision patient positioning devices and a fundamental shift in our understanding of the radiobiology of high dose radiation, Stereotactic Body Radiotherapy (SBRT) has emerged for the treatment of spinal metastases.

SBRT achieves local tumor control rates exceeding 90% in early stage non-small cell lung cancer (NSCLC). The methodology of image-guided SBRT was transferred from lung cancer to vertebral metastases aiming at more rapid and especially long-term pain and tumor control by more intense irradiation [5]. Spine SBRT was quickly adopted in the radiotherapy community [6]. However, this broad clinical implementation is supported by only few prospective trials [7,8]: evidence is mostly based on small, retrospective, and single-institution analyses.

Although the risk of radiation induced myelopathy is low after spine SBRT [9,10], unexpectedly high rates of “new” toxicities like vertebral compression fracture have been described [11]. These observations combined with a lack of standardization of spine SBRT practice indicate that larger studies with longer follow-up as well as prospective trials are required to establish the methodology and value of SBRT in the multidisciplinary management of spinal metastases. Therefore, it was the aim of this study to establish a multi-institutional database of spine SBRT and to analyze patient selection criteria, methodology, safety and clinical outcome after spine SBRT.

## Materials and methods

Eight international centers from the United States (n = 5), Canada (n = 2) and Germany (n = 1) participated in this retrospective study. The local ethics committee approved participation in this study in all eight centers. The study is based on 301 patients treated for 387 vertebral metastases (11 to 118 per institution) between 2004 and 2013; 370 of 387 SBRT treatments were performed 2008 and later.

A homogeneous patient cohort was analyzed in this study: SBRT was used as re-irradiation in none of the cases and no patient suffered from symptomatic spinal cord compression. All centers are members of the “Elekta Spine SBRT Research Consortium” and therefore, identical treatment delivery technology was used in all treatments. Patients were treated with linac based SBRT using daily cone-beam CT based image-guidance, online correction of set-up errors in six degrees of freedom using the robotic HexaPod™ couch (Elekta AB, Stockholm, Sweden) and intensity modulated radiotherapy (IMRT) was delivered using a multileaf collimator with 4 mm leaf width (BeamModulator™, Elekta AB, Stockholm, Sweden).

Other details of treatment planning and delivery were not standardized between institutions and will therefore be presented in the results part of the manuscript.

In order to correlate irradiation doses with clinical results, biological equivalent doses in 2 Gy fractions (EQD2) were calculated: an α/β-ratio of 10 Gy was assumed for spinal metastases and an α/β-ratio of 2 Gy for the spinal cord. The EQD2 was calculated using the linear quadratic model (n = number of fractions; d = single fraction dose):

$$ EQD2(Gy)=n*d*\left(d + \alpha /\beta \right)/\left(2+\alpha /\beta \right) $$

Imaging (CT, MRI or FDG-PET CT) was required for assessment of local tumor control (LC) and local failure was defined as tumor regrowth in the treated volume according to institutional protocol. Progressive clinical symptoms or pain without local tumor recurrence in imaging were not sufficient for definition of local failure.

Pain at the treated vertebral level was categorized into pain-free, mild-to-moderate pain and severe pain prior to SBRT and at the last follow-up. If detailed information as the visual analog scale was available, pain-free, mild-to-moderate pain and severe pain were equivalent to scores of 0, 1-5 and 6-10, respectively.

Statistical analyses were performed with Statistica X (Statsoft, Tulsa OK), and all statistical tests were two-sided. OS was evaluated per patient and all other endpoints per SBRT treatment. The Pearson chi-square/Fisher’s Exact test and Kruskal-Wallis ANOVA were used to compare categorical and continuous variables between groups, respectively. Receiver Operating Characteristics (ROC) curves were used to test prognostic factors (irradiation dose) in predicting outcome, with their performances measured based on the area under the ROC curve. Estimated likelihood of events was calculated using the Kaplan Meier method with start of follow-up on the last day of SBRT treatment. The log-rank test was used to compare differences between curves in univariate analysis. Multivariate analysis was performed using Cox-proportial Hazard method with backward exclusion of non-significant variables; all variables, which were statistically significant in the univariate analysis, were included into the multivariate analysis. A p-value of ≤0.05 was considered statistically significant.

## Results

### Patient and treatment characteristics

**Figure 1:**
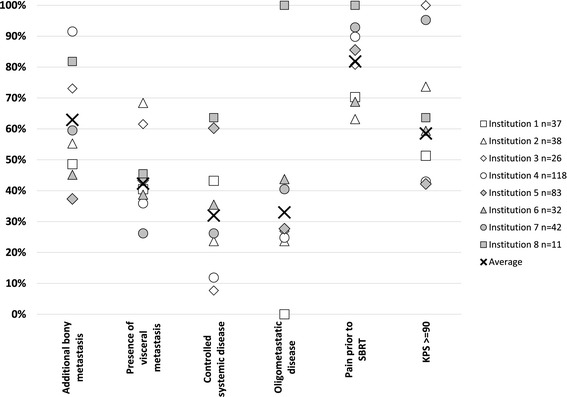
**Inter-institutional variability in patient characteristics.**

Patient characteristics and characteristics of the treated vertebral metastases are listed in Table 1. Inter-institutional variability in patient selection criteria is illustrated in Figure 1. The majority of patients presented with a good performance status prior to SBRT (median Karnofsky performance status 90). About one quarter of the patients were treated for a solitary vertebral metastasis without further evidence of malignancy; additional bone and visceral metastases were present in 62.9% and 42.3% of the patients, respectively. Patients were free from epidural disease extension in 41.8% (Bilsky score 0). Lesions had an osteolytic component in 72.3% resulting in a preexisting compression fracture rate of the treated vertebra in 19.8%.

**Table 1:** **Patient (n = 301) and lesion (n = 387) characteristics; percentages are given per SBRT treatment**

**Characteristic**	**Median**	**Range**	**Proportion (%)**
Age (years)	61.3	9 – 91	
Performance status	90	40 – 100	
Sex (male)			55.1
Interval PD to SBRT (years)	2.5	0 – 41	
Primary disease			
Breast			20
RCC			19
NSCLC			16
Other			45
Pain prior to SBRT			
No			18.2
Yes			81.8
Solitary metastasis (yes)			23.0
Systemic disease considered as controlled prior to SBRT (yes)			32.0
Cancer treated with curative intend at primary diagnosis (yes)			67.7
Additional bone metastasis (present)			62.9
Visceral metastases (present)			42.3
Bilsky Score 0 (yes)			41.8
Paraspinal involvement of spine metastasis (yes)			44.8
Osteolytic component of spine metastasis (yes)			72.3
Compression fracture of spine metastasis (yes)			19.8

**Figure 2:**
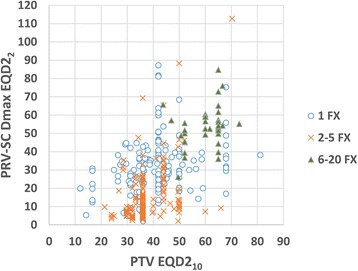
**Distribution and correlation between PTV prescription dose (EQD2**_**10**_ **) and maximum dose to the PRV-SC (EQD2**_**2**_ **); results are shown for SBRT delivered in 1 fraction (1 FX), 2-5 fractions (2-5 FX) and 6-20 fractions (6-20 FX).**

Treatment characteristics are summarized in Table 2. A dedicated MRI and FDG-PET were used for target and organ-at-risk definition in 85% and 18%, respectively. Patients were immobilized using the BodyFix™ (Elekta AB, Stockholm, Sweden) system (80.6%), a thermoplastic mask (11.9%), a combination of both (3.5%) or other devices (4%). Step-and-shoot IMRT was practiced in 96% of all cases, and an anatomical target volume concept was used in the majority of the cases (61%) [12]. A median of one vertebrae (1-6) in one single target volume was treated with median 3 irradiation fractions (1-20) to a median prescription dose of 24 Gy (10 – 60 Gy). The spinal cord, thecal sac and spinal canal formed the basis for generation of the planning risk volume (PRV-SC) in 33.4%, 22.3% and 42.3%, respectively, and a PRV margin of 1-2 mm was used in 85% of the cases. The maximum point dose to the PRV-SC was median 10 Gy (maximum 65 Gy). After calculation of 2 Gy equivalent doses, 90% of all SBRT treatments were performed with prescription PTV doses and maximum PRV-SC doses of 27-65 Gy (EQD2_10_) and 5-59 Gy (EQD2_2_), respectively. Variability of doses to the PTV and PRV-SC and the association between PTV and PRV-SC doses are illustrated in Figure 2.

**Table 2:** **SBRT treatment (n = 387) characteristics**

**Characteristic**	**Median**	**Range**	**Proportion (%)**
Number of vertebras treated in one target volume	1	1 – 6	
Number of vertebras treated in one target volume n = 1			70.4
Number of vertebras treated in one target volume n = 2–3			24.7
Number of vertebras treated in one target volume n = 4–6			4.9
Treatment fractions	3	1 – 20	
Treatment fractions n = 1			39.5
Treatment fractions n = 2–5			51.4
Treatment fractions n = 6–20			9.1
Prescription dose (Gy)	24	8 – 60	
PTV (cm^3^)	34	0.8 – 721	
Max. point dose PRV spinal cord	10	2 – 65	
Prescription dose (EQD2/10 Gy)	37.7	12 – 81	
Max. point dose PRV spinal cord (EQD2/2 Gy)	22.4	2 – 112

### Clinical outcome in the total patient population

**Figure 3:**
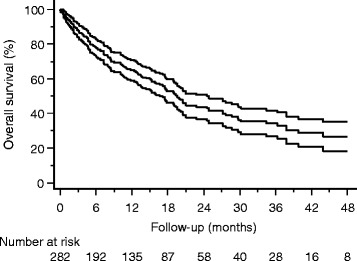
**Overall survival analyzed per patient: Kaplan Meier Curve with 95% confidence interval.**

The median follow-up was 11.8 months (0-105 months), median OS was 19.5 months; one-year and two-years OS were 64.9% and 43.7% (Figure 3), respectively. Univariate analysis was performed for patient and treatment factors associated with OS and significant parameters were included into multivariate analysis. The following characteristics were significantly associated with worse OS: male sex (p < 0.001; HR = 0.44), performance status <90 (p < 0.001; HR = 0.46), presence of visceral metastases (p = 0.007; HR = 0.50), uncontrolled systemic disease (p = 0.007; HR = 0.45), >1 vertebra treated with SBRT (p = 0.04; HR = 0.62).

**Figure 4:**
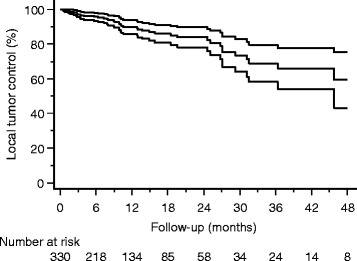
**Local tumor control analyzed per treated lesion: Kaplan Meier Curve with 95% confidence interval.**

LC was assessed using CT (25.4%), MRI (63.2%), FDG PET (1.2%) or FDG PET-CT (10.2%); follow-up was too short for analysis of local tumor control in 15%. One-year and two-years LC were 89.9% and 83.9% (Figure 4), respectively. Median time to development of local failure was 9 months (1 – 55 months).

Univariate analysis was performed for patient and treatment factors associated with local tumor control and significant parameters were included into multivariate analysis. The following characteristics were significantly associated with worse outcome: interval between primary diagnosis of cancer and SBRT of ≤30 months (p = 0.01; HR = 0.27) and histology of primary disease (NSCLC, renal cell cancer, melanoma, other) (p = 0.01; HR = 0.21).

**Figure 5:**
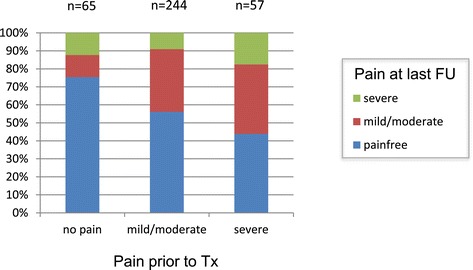
**Pain response depending on the pre-SBRT pain score.**

Prior to SBRT, patients were pain-free, suffered from mild/moderate pain and severe pain in 18.2%, 64.9% and 16.9%, respectively. Detailed pain response at the treated spinal level after a median follow-up of 11.5 months is illustrated in Figure 5. Patients being pain-free, suffering from mild/moderate and severe pain prior to SBRT were pain-free at the time of the last clinical assessment in 76.8%, 56.3% and 43.8%, respectively. After uni- and multivariate analyses, no patient or treatment characteristic was significantly associated with improved pain control.

Acute toxicity was mild in the majority of the patients: grade 3 toxicity was observed in only 2 patients (Table 3). Median follow-up for assessment of long-term toxicity was 11.5 months, maximum 105 months. Progressive of new vertebral compression fractures were diagnosed in 30 cases (7.8%), of which the fracture was newly developed after SBRT in 14 cases (3.6%) and progressive after SBRT in 16 cases (4.1%). No case of radiation-induced myelopathy (RIM) was observed.

**Table 3:** **Absolute number of patients where acute toxicity was assessed and proportion of patients with grade 0-3 toxicity**

	**Dermatitis**	**Dysphagia**	**Pain**
Toxicity assessment available (n)	281	283	304
Grade 0 (%)	96.1	89.8	83.9
Grade 1 (%)	3.9	9.5	9.6
Grade 2 (%)	0	0.7	5.9
Grade 3 (%)	0	0	0.7

### Comparison of patients treated with 1-5 and 6-20 SBRT fractions

Patient characteristics and clinical outcome were compared between SBRT with 1-5 fractions (n = 352) and hypo-fractionated SBRT with 6-20 fractions (n = 35). Hypo-fractionated SBRT was practiced in patients, who had received palliative chemotherapy prior to SBRT less frequently (17% vs 61%; p < 0.001) and who suffered from pain less frequently (69% vs 84%; p = 0.03); all other patient characteristics were not statistically different. Metastatic lesions treated with hypo-fractionated SBRT were osteolytic more frequently (94% vs 70%; p = 0.001), were characterized by a compression fracture prior to SBRT more frequently (37% vs 18%; p = 0.01) and had a distribution with higher Bilsky scores (p < 0.001). Irradiation doses were significantly higher in patients treated with hypo-fractionated SBRT: EQD2_10_ PTV prescription doses were 60 Gy and 40 Gy on average (p < 0.001), respectively and EQD2_2_ maximal PRV spinal cord doses were 54 Gy and 23 Gy (p < 0.001), respectively. In terms of clinical outcome, median OS was 19 months and 20 months after SBRT with <5 and 6-20 SBRT fractions (p = 0.89), respectively. Local tumor was not different as well: two year LC was 84% and 82% (p = 0.4), respectively. The development of new fractures after SBRT was not correlated with the number of SBRT fractions.

## Discussion

This is the largest study reporting detailed data regarding patient selection criteria, clinical practice and outcome of spine SBRT in a multi-institutional environment. Substantial variability was observed in major aspects of spine SBRT, despite the fact that nearly identical linac-based SBRT technologies were utilized at all eight participating institutions. This inter-institutional variability is most likely explained by similar variability of SBRT practice in the literature and the lack of clearly established patient selection criteria and practice guidelines [13]. Results of our study may therefore be considered as representative for current spine SBRT practice in the radiotherapy community.

Whereas some centers explicitly selected patients with long life expectancy for their spine SBRT practice, this was not done by other institutions. The proportion of patients free from visceral metastases prior to SBRT ranged between 31.6% and 73.8% and oligomatastatic disease was stated as the primary reason for SBRT in between 23.7% and 100% of institutional cases. Similar variability was observed for relevant treatment characteristics. Fractionation ranged from single-fraction radiosurgery to hypo-fractionated SBRT; one institution treated the majority of their cases with 20 fractions, which is not considered as SBRT based on the US SBRT definition. However, treatment was planned and delivered with identical accuracy as single fraction radiosurgery and the fractionation of 20 × 3 Gy equates to a radiosurgical dose of >23 Gy rendering this approach highly biologically active [14].

Despite this variability in patient and treatment characteristics, toxicity was minimal in this study. Most importantly, no single case of RIM was observed. The average time to development of RIM after conventional radiotherapy is approximately 18 months [15]. Gibbs et al. reported the largest study of six RIM cases in 1075 spine SBRT cases [9]: their 6 months time interval to development of RIM appears shorter compared to conventional radiotherapy. Follow-up was longer than 6 months and 18 months in 192 and 87 of our patients, respectively, indicating a sufficiently long follow-up for reliable analysis of RIM. The absence of RIM in our study is especially encouraging as epidural disease with a Bilsky score of >0 was present in 58% of our patients [16]. The absence of RIM maybe explained by several safety measures, which were consistently practiced in our patient cohort: 1) use of the PRV concept for the spinal cord, 2) maximum EQD2_2_ doses to the PRV spinal cord ≤60 Gy in >95% of the cases, 3) daily volumetric image-guidance with online correction of set-up errors in six degrees of freedom and 4) use of customized patient immobilization for minimization of intra-fractional patient motion. These measures might therefore be recommended for safe practice of spine SBRT.

Vertebral compression fractures were observed in only 7.8% of the treatments and half of them were progressive fractures, which existed *prior* to SBRT. Compression fractures are more frequently described in the literature with rates of 11-39% [11,17,18]. Based upon these limited data available, very high single fraction doses >20 Gy appear to be associated with compression fractions, and this might explain the low rate of toxicity in the current study: only 22/387 cases were treated with such high single fraction doses.

There is some controversy whether or not the increased cost and workload associated with SBRT is appropriate in the palliative setting of vertebral metastases [19]. Indeed, OS is short in unselected patients treated with conventional radiotherapy for painful vertebral metastases: Mizimoto et al. reported a median OS of only 5.9 months in 544 patients [20] and van der Linden reported a median OS of 7 months in a cohort of 342 patients, who were randomized between a single fraction of 8 Gy and 6 × 4 Gy [21].

However, a substantially longer OS of median 19.5 months was observed in the current study. Based on the fact that conventional radiotherapy achieves pain control only for the short duration of half a year [22–25], the majority of the patients in this study were at risk for recurring pain if conventional radiotherapy would have been performed. It is also important to put this favorable overall survival rate into the perspective of locally advanced NSCLC for example, where median OS is of similar magnitude despite aggressive multimodal treatment with curative intent [26,27].

OS varies substantially between studies using SBRT for treatment of vertebral metastases. Favorable OS was reported by Wang et al. with a median OS of 23 months in 149 patients, where SBRT was used as primary treatment and re-irradiation in a prospective phase II study [7]. Median OS was as long as 30 months in 61 patients treated with single fraction radiosurgery in a phase I/II study [28]. Whether the favorable OS in this study and the studies above is a result of the applied patient selection criteria or whether SBRT in an oligometastatic disease setting contributes to prolonged OS remains to be evaluated. In contrast, Amdur et al. reported a 1-year OS of only 25% in 25 patients treated within a phase II study of single fraction radiosurgery [29]; Schipani et al. reported a median OS of 8 months [30], Heron et al. reported a median OS of 13 months [31]. A recursive partitioning analysis to predict OS was performed by Chao et al., and the three factors of age, Karnofsky performance status and the time from primary diagnosis to SBRT allowed differentiation of median OS between 2.4 months and 21.1 months [32]. In our study, mulitvariate analysis identified several clinical parameters, which were correlated with OS: gender, performance status, presence of visceral metastases, uncontrolled systemic disease, number of involved vertebras. These factors might help in the selection of patients with long life expectancy for SBRT, but validation is required.

In this context of favorable OS, long-term local tumor control was observed with actuarial rates of 89.9% and 83.9% at one and two years, respectively. This is in agreement with other reports of spine SBRT, where long-term local tumor control was achieved in >80% of the cases [28,31,33–37]. Histology was significantly correlated with local tumor control and worse outcome was observed in histologies known to be less radiosensive: NSCLC, renal cell cancer and melanoma. A similar correlation was described by Heron et al. [31] but not by other studies [28,33].

A dose-response relationship was expected for achievement of LC, but such correlation was not observed. Prescribed physical doses and biological effective doses were not correlated with LC. Additionally, maximum doses to the PRV spinal cord and pre-SBRT Bilsky score were not significant as well, factors which should be correlated with minimum PTV doses [33,38]. Several but not all studies [28,34] reported that higher SBRT do result in better LC, but there is no consensus about the detailed dose and fractionation. Laufer et al. reported improved outcome after high-dose (median total dose 27 Gy in 3 fractions) compared to low dose SBRT (median total dose 30 Gy in 5 or 6 fractions) [33]. Al-Omair et al. described better LC after 18–26 Gy in 1–2 fractions compared to 18–40 Gy in 3–5 fractions [38]. In contrast, LC was better after multiple-fraction compared to single fraction SBRT in the study by Heron et al. [31]. Lovelock et al. not only evaluated prescribed doses but performed a more detailed dosimetrical analysis and minimum PTV doses >15 Gy were significantly associated with better LC [35]. Based on potential variability in the method of dose prescription in this multicenter study, a detailed dosimetrical analysis similar to Lovelock et al. is currently underway. Additionally, radiological assessment of local tumor control or tumor recurrence is difficult in many cases and has been analyzed systematically only very recently [39]; this lack of established criteria for local tumor control might influence our multicenter analysis as well.

Finally, long-term LC was found to be associated with long-term pain control. The high rates of complete pain response ranging between 77% and 44% (depending on the pre-SBRT pain score) appear promising compared to complete pain response rates of only 25-40% after conventional palliative radiotherapy [22–25]. Unfortunately, the retrospective nature of this study did not allow for a longitudinal pain assessment as well as analysis of pain medication.

Strengths of this study include the large number of 387 SBRT treatments performed at eight experienced international centers. A homogeneous patient cohort of primary SBRT excluding re-irradiated patients and excluding patients treated for symptomatic spinal cord compression was analyzed. All patients were treated with linac-based SBRT using identical equipment. Follow-up was sufficiently long with a median of 11.7 months. Weaknesses of this study are the retrospective nature of our multi-institutional analysis.

## Conclusions

Linac based SBRT for vertebral metastases was determined to be safe in this multi-institutional environment with no case of radiation-induced myelopathy. Use of the PRV concept for the spinal cord, maximum EQD2_2_ doses to the PRV spinal cord ≤60 Gy, daily volumetric image-guidance with online correction of set-up errors in six degrees of freedom and the use of customized patient immobilization are recommended measures for safe SBRT practice. In a patient cohort with favorable OS, SBRT achieved high rates of long-term local tumor control, which appears better compared to conventional radiotherapy alone.
